# Efficacy of Fine-Tuned Large Language Model in CT Protocol Assignment as Clinical Decision-Supporting System

**DOI:** 10.1007/s10278-025-01433-6

**Published:** 2025-02-05

**Authors:** Noriko Kanemaru, Koichiro Yasaka, Naomasa Okimoto, Mai Sato, Takuto Nomura, Yuichi Morita, Akira Katayama, Shigeru Kiryu, Osamu Abe

**Affiliations:** 1 Department of Radiology, Graduate School of Medicine, The University of Tokyo, 7-3-1 Hongo, Bunkyo-Ku, Tokyo, 113-8655 Japan; 2 Department of Radiology, International University of Health and Welfare Narita Hospital, 852 Hatakeda Narita, Chiba, 286-0124 Japan

**Keywords:** Large language model, CT protocol classification, Deep learning

## Abstract

Accurate CT protocol assignment is crucial for optimizing medical imaging procedures. The integration of large language models (LLMs) may be helpful, but its efficacy as a clinical decision support system for protocoling tasks remains unknown. This study aimed to develop and evaluate fine-tuned LLM specifically designed for CT protocoling, as well as assess its performance, both standalone and in concurrent use, in terms of effectiveness and efficiency within radiological workflows. This retrospective study included radiology tests for contrast-enhanced chest and abdominal CT examinations (2829/498/941 for training/validation/testing). Inputs involve the clinical indication section, age, and anatomic coverage. The LLM was fine-tuned for 15 epochs, selecting the best model by macro sensitivity in validation. Performance was then evaluated on 800 randomly selected cases from the test dataset. Two radiology residents and two radiologists assigned CT protocols with and without referencing the output of LLM to evaluate its efficacy as a clinical decision support system. The LLM exhibited high accuracy metrics, with top-1 and top-2 accuracies of 0.923 and 0.963, respectively, and a macro sensitivity of 0.907. It processed each case in an average of 0.39 s. The LLM, as a clinical decision support tool, improved accuracy both for residents (0.913 vs. 0.936) and radiologists (0.920 vs. 0.926 without and with LLM, respectively), with the improvement for residents being statistically significant (*p* = 0.02). Additionally, it reduced reading times by 14% for residents and 12% for radiologists. These results indicate the potential of LLMs to improve CT protocoling efficiency and diagnostic accuracy in radiological practice.

**Supplementary Information:**

The online version contains supplementary material available at 10.1007/s10278-025-01433-6.

## Background

Today’s large language models (LLMs) have demonstrated promising results in various tasks, including several radiology applications [1–4]. The CT protocol assignment task is one specific area where LLMs could be particularly beneficial [5–7].

Radiologists or radiology technologists manually assign CT protocols to ensure that exams are appropriately conducted for each CT imaging procedure. This assignment is crucial to maximize the contribution of imaging to patient care while limiting unnecessary radiation exposure. However, selecting the most suitable protocol for a specific patient and situation from a variety of complex protocols (often including institution-specific ones) is complex, even for experienced radiologists, and is prone to errors [8]. Additionally, a previous study reported that protocol assignment is time-consuming, occupying 17% of the non-imaging interpretative tasks of radiologists [9]. LLMs have the potential to address these issues by reducing protocoling errors and decreasing the workload for clinical staff and radiologists.

Previous studies have investigated various algorithms for CT or MRI imaging protocol assignment, including support vector machine, naive Bayes, random forest, gradient boosted machines, k-nearest neighbors, fastTEXT, convolutional neural networks, long short time memory [5–7, 10–17], and LLMs, specifically bidirectional encoder representations of transformers (BERT) [5–7, 14]. Notably, BERT’s deep contextual word embeddings have significantly improved text classification and have achieved accuracy between 0.922 and 0.93 [5, 14] and F1 scores between 0.84 and 0.901[6, 7]. Additionally, BERT outperforms support vector machines and naive Bayes on minority classes, making it especially beneficial in protocol classification, where data are often highly imbalanced [6]. Furthermore, BERT models are fine-tuned as institution-specific with relatively small datasets; thus, their customizability and flexibility improve their applicability [18].

However, the current models have not yet reached a feasible full automation level. To date, their clinical application is limited to a clinical decision support system that still requires concurrent use with radiologists, given the task’s high-stakes nature. While a system presenting a concise list of protocols, often including the most appropriate one, can facilitate protocol assignment, it may also introduce potential biases, such as default bias (accepting an incorrect recommendation) and overconfidence bias (ignoring a correct recommendation) [19]. These systems can also reduce efficiency by requiring additional consideration of the model’s recommendations. Although some studies have compared the model’s performance with radiologists’ manual labeling [13, 14], none has directly compared the performance of radiologists’ protocol assignments with and without the aid of automated models. Therefore, the effect of implementing such models as clinical decision support systems on radiology workflow remains unclear. Furthermore, no studies have assessed the impact of these systems based on the radiologists’ experience level.

This study aims to evaluate the efficacy of a fine-tuned BERT model in CT protocol assignment in radiological workflow. We will establish a BERT model tailored for institution-specific CT protocols and compare its effectiveness (accuracy) and efficiency (review time) when used concurrently by radiologists. Additionally, we will assess the association of the model’s effectiveness with the radiologist’s experience level.

## Materials and Methods

Our Institutional Review Board approved this retrospective study and waived the requirement for obtaining written informed consent from patients because of the retrospective study design.

### Datasets

**Fig. 1:**
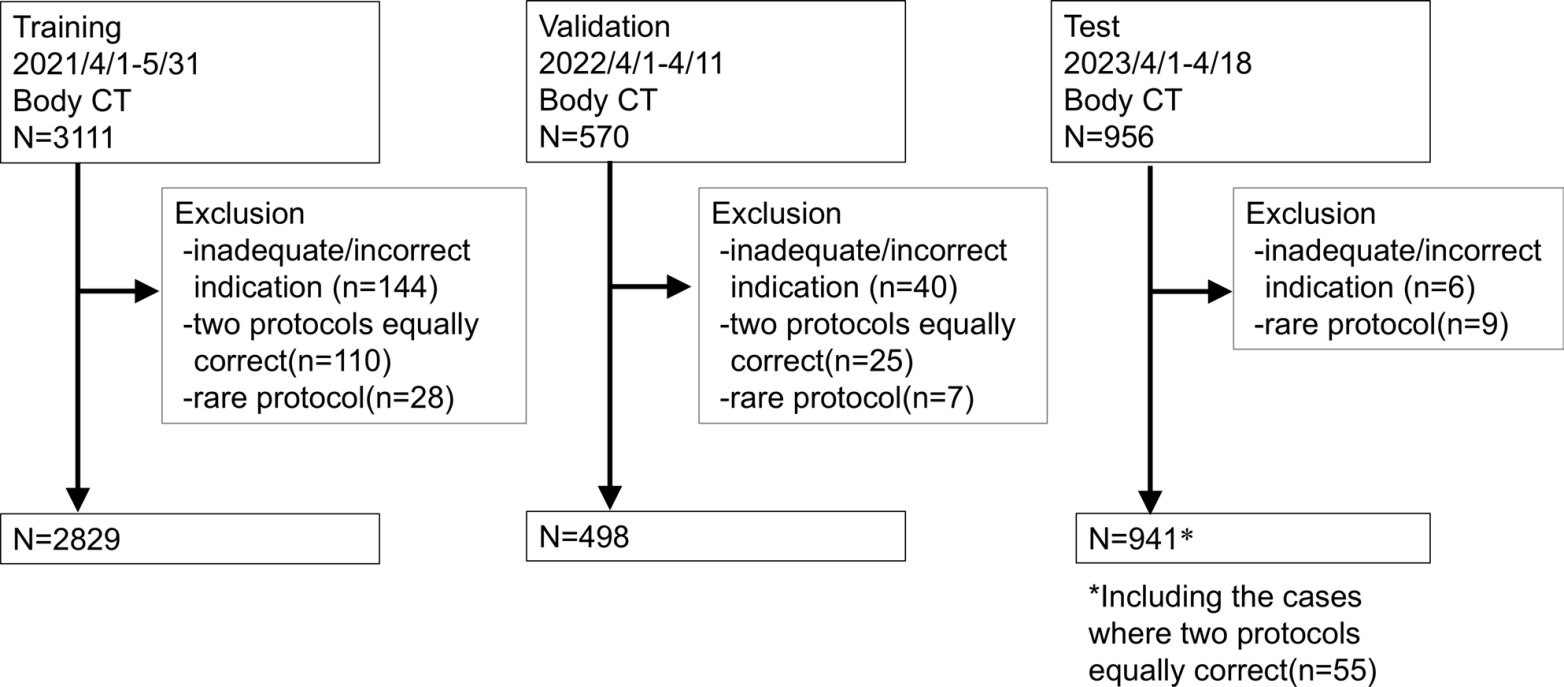
Data selection for training, validation, and test dataset

The training, validation, and test datasets included contrast-enhanced chest and abdominal CT examinations conducted from April 1 to May 31, 2021, from April 1 to 11, 2022, and from April 1 to 18, 2023, respectively. In our hospital, many doctors are replaced on a yearly basis, leading to variations in clinical indications depending on the referring physician each year. This results in dataset variations across different years. By using a temporally independent dataset (i.e., three different years for the training, validation, and test datasets), we were able to evaluate the model’s performance in light of these variations and ensure its robustness [20]. Clinical indications, age, and anatomic coverage of the exams written in Japanese were collected from the Picture Archiving Communicating System and saved in CSV format. Of 3111, 570, and 956 radiology exam requests, 144, 40, and 6 were excluded due to inadequate or inappropriate clinical indications to assign protocols in the training, validation, and test datasets, respectively. We included only protocols with frequencies of at least 0.3%, narrowing them down to 12 protocols (Table 1), due to the rarity of protocols mainly used for research studies or uncommon clinical scenarios. This adjustment resulted in 2939, 523, and 941 requests for the training, validation, and test datasets, respectively. Cases where two protocols were assumed to be uniquely optimal were excluded from the training and validation datasets (110/25 cases) but included in the test dataset (55 cases) by accepting two true labels, to closely simulate the real-world tasks in the test dataset. Finally, the final training, validation, and test datasets consisted of 2829, 498, and 941 requests, respectively (Fig. 1). Table 2 shows the label frequency of each category in the training, validation, and test datasets.

**Table 1:** CT protocol classes

Protocol name	Protocol description
Hepatic mass	Five-phase liver protocol
Liver donor	Four-phase liver protocol for donor candidate
Acute abdomen	Three-phase abdominal protocol for acute abdominal pain
Stent graft	Three-phase protocol for endovascular stent grafting
CT angiography	CT angiography with or without unenhanced and late-phase
Routine	One-late phase with or without drink
Urinary tract	Three-phase protocol including urogram
Renal mass	Three-phase renal protocol
Pancreas/biliary	Four-phase pancreatic protocol
Colorectal	Two-phase protocol for colon cancer pre-operative assessment
CT pulmonary angiography	Two-phase protocol for pulmonary embolism with or without late-phase
Bronchial artery	Two-phase protocol for esophageal cancer pre-operative evaluation

**Table 2:** Patient background information and prevalence of each protocol in training, validation, and test datasets

	Training	Validation	Test
Number of reports	2829	498	941
Age (years ± standard deviation)	63.4 ± 15.6	64.8 ± 14.6	64.0 ± 14.8
Sex (male/female)	1619/1210	291/207	548/393
Protocol			
Hepatic mass	442	67	176 (19)
Liver donor	23	7	7
Acute abdomen	74	12	35 (9)
Stent graft	15	1	6
CT angiography	159	18	57 (15)
Routine	1550	276	500 (47)
Urinary tract	50	11	6 (3)
Renal mass	25	4	9
Pancreas/biliary	309	66	102 (16)
Colorectal	61	14	9
CT pulmonary angiography	111	20	30 (1)
Bronchial artery	10	2	4

The parentheses indicate the number of the data in which two labels were accepted as true labels

### Reference Standard

The clinical indication section, age, and anatomic coverage of the exam were reviewed. Radiologist A, with imaging experience of 4 years, conducted these evaluations for the training and validation datasets. Radiologists A and B (with imaging experience of 6 years) established the reference standard for the test dataset. In cases of disagreement, the true label was determined by senior radiologist C, who has 14 years of imaging experience.

### Fine-Tuning of the Pretrained LLM

**Fig. 2:**
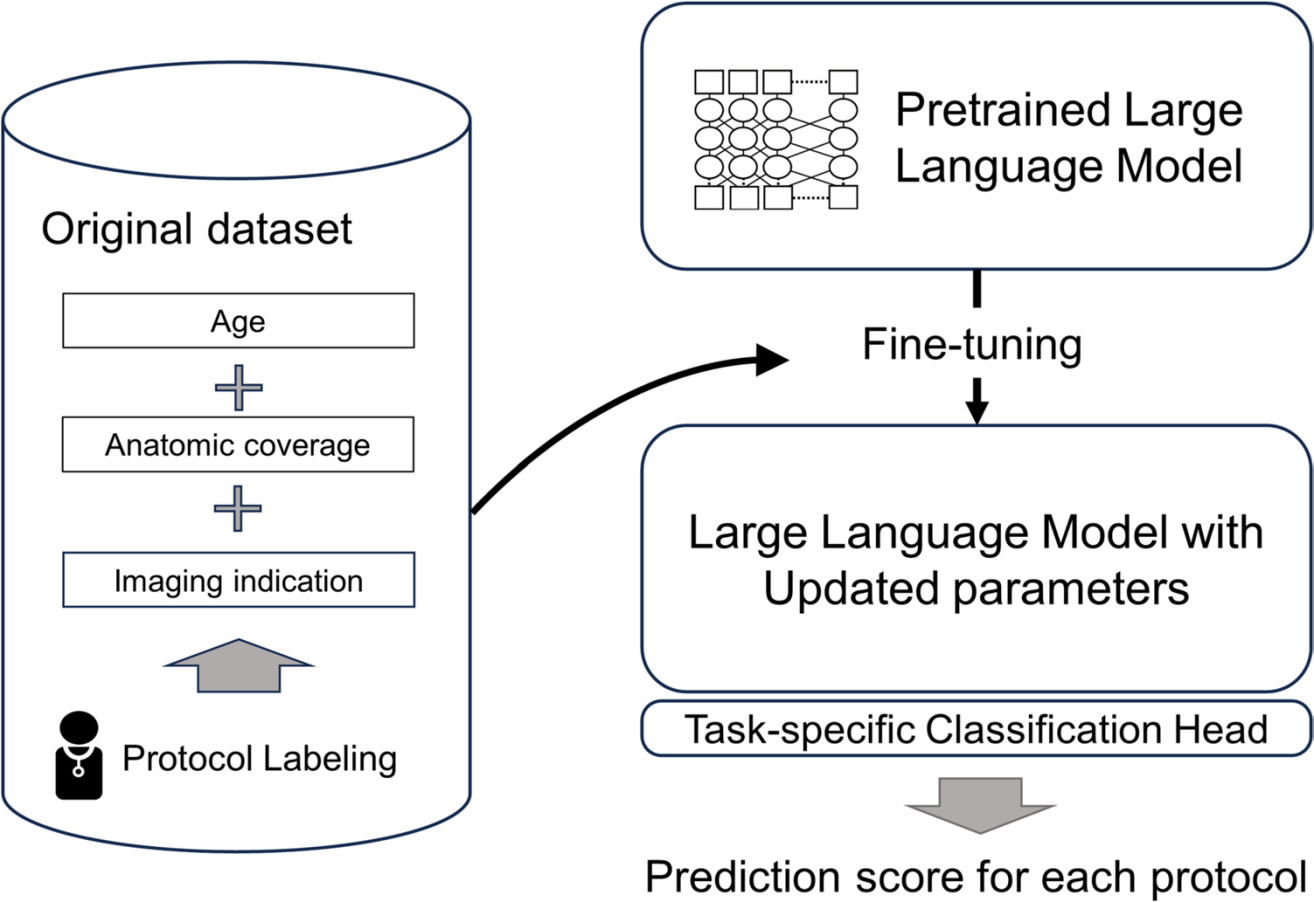
The process of fine-tuning a pre-trained large language model for CT protocol classification. The original dataset comprises patient age, anatomic coverage, and clinical indication, which are concatenated into single strings to serve as input data. Corresponding protocol assignments are used as labels for training. This combined dataset is fed into the pretrained language model, where fine-tuning updates its parameters to better suit the specific task. Finally, a task-specific classification head is attached to the fine-tuned model to generate prediction scores for each CT protocol based on the input data

Programming language of Python version 3.10.13 (https://www.python.org/) and Transformers library version 4.35.2 (https://huggingface.co/) on a workstation equipped with a central processing unit of Core™ i9-10980XE, a graphic processing unit of GeForce RTX™ 3060 (NVIDIA), and a random access memory of 64 GB were utilized for fine-tuning the pretrained BERT Japanese model (https://huggingface.co/cl-tohoku/bert-base-japanese). The model, including 12 layers, 768 hidden states dimensions, and 12 attention heads, was pretrained on the Japanese Wikipedia as of September 1, 2019. The text data was tokenized using the BERT tokenizer, converting the input into IDs, token type IDs, and attention masks. Input involved a single string concatenated from the sections of clinical indication, age, and anatomic coverage, categorized into 12 groups according to logits for each group (Fig. 2). Fine-tuning and validation were conducted in five sessions, each using a differently shuffled training dataset seed, with 15 epochs per session, to consider variability in fine-tuning results and select the best-performing model. We adjusted the loss function by setting the weight of each class proportional to the inverse of its prevalence in order to address class imbalance [21, 22]. Other hyperparameters were set and fixed at their default values as described in the Transformers library (https://huggingface.co/docs/transformers/main_classes/trainer). The model with the best macro sensitivity (calculated by averaging the sensitivities of each class with equal weight given to each class) in the validation dataset was saved and further assessed in the following session. The code used for fine-tuning is provided in the supplemental material.

### Evaluation of the Fine-Tuned LLM in the Test Dataset

The best-performing model was examined on a randomly selected subset of 800 cases from the test dataset, which was also used in the subsequent evaluation section involving the radiologists. (The number was limited to 800 cases to mitigate reader fatigue, while the remaining 141 cases were allocated for practice sessions as described below.) The evaluation included reviewing time, weighted accuracy, and macro sensitivity.

### Evaluation of the Fine-Tuned LLM as Clinical Decision Support System

In Japan, a 2-year clinical internship involves rotations across multiple specialties after graduation. Afterward, a 3-year radiology residency program is required, followed by 2 years of subspecialty fellowship training. Readers were recruited from two groups to assess the effect of fine-tuned LLM across different stages of training. The first group consisted of radiology residents in the residency program (readers 1 and 2, with a total of 2 years post-graduate experience, including 3 months of imaging experience, also known as “radiology resident”). The second group involved general radiologists in subspeciality fellowship training (readers 3 and 4, with a total of 5 years of post-graduate experience, including 3 years of imaging experience, referred to as “radiologists”).

The readers’ performance was categorized into the radiologists and radiology resident groups and then combined.

**Fig. 3:**
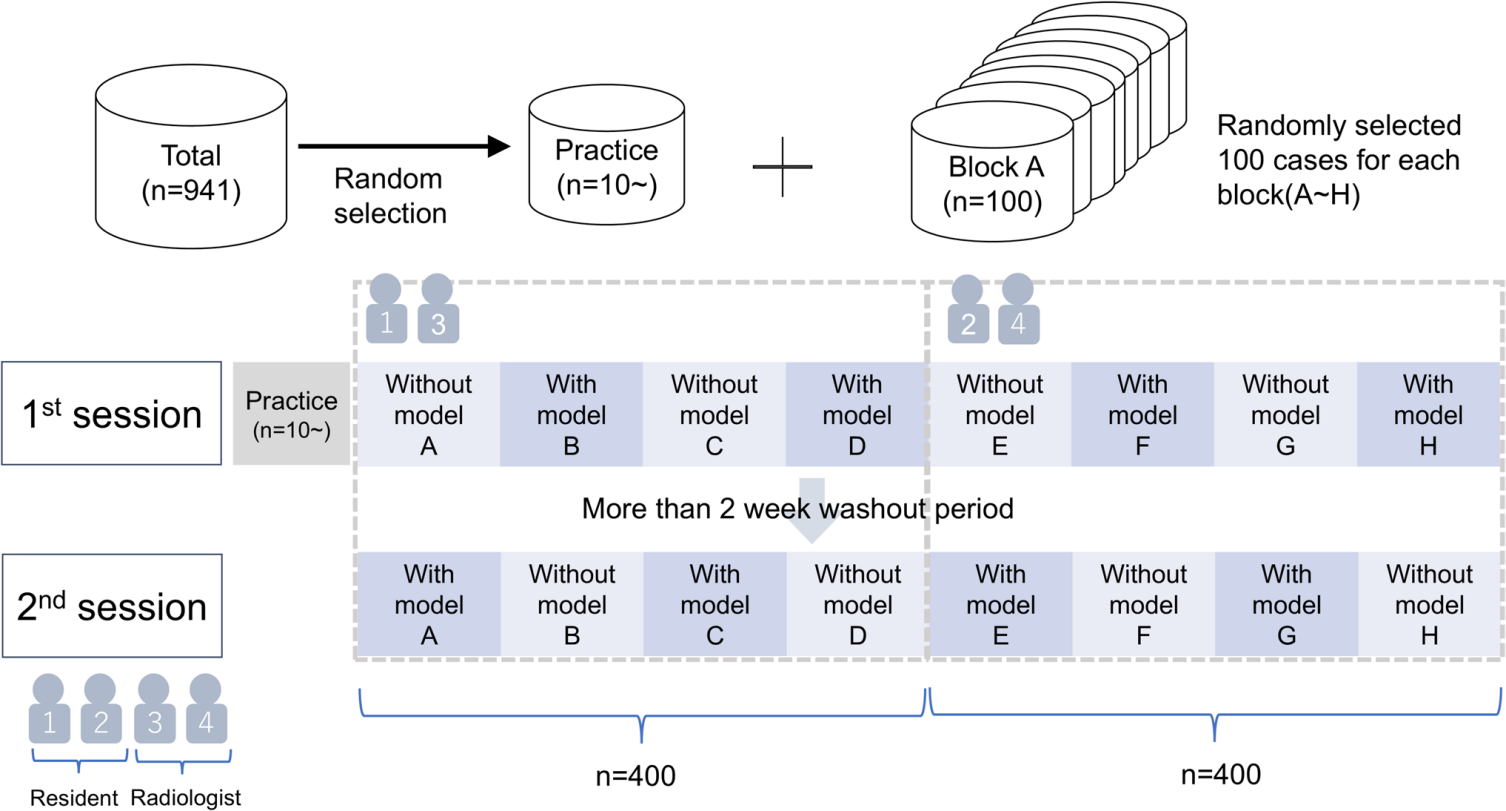
Schematic of observer performance test. From the 941 test datasets, 8 blocks (each containing 100 cases) were randomly selected (blocks A–H). Among the 4 observers, 1 radiologist and 1 radiology resident (readers 1 and 3) reviewed blocks A–D, whereas the other radiologist and radiology resident (readers 2 and 4) reviewed blocks E–H. Each observer reviewed each case twice over two reading sessions. The order of using assistance and non-assistance was changed per block. A washout period of at least 2 weeks was set between the two sessions. A practice round was performed using the remaining cases with and without assistance before the main evaluation

The cases per session were limited to 400 cases for each observer because too many samples and test duration caused the reader’s fatigue and deteriorated the quality of collected data. A total of 8 blocks (each containing 100 cases) were randomly selected (blocks A–H) from the 941 test datasets. Among the 4 observers, 1 radiologist and 1 radiology resident (readers 1 and 3) reviewed blocks A–D, whereas the other radiologist and radiology resident (readers 2 and 4) reviewed blocks E–H. To reduce bias, readers reviewed 200 and another 200 cases without and with the fine-tuned LLM, respectively, during one session and complementary cases during a second session so that each observer reviewed each case twice: once without and once with the fine-tuned LLM. The order of using assistance and non-assistance was alternated per block. A washout period of at least 2 weeks was set between the two sessions to minimize recall bias. A practice round was performed using the remaining cases with and without assistance before the main evaluation. Each reader was required to practice with and without assistance on at least 10 cases, with a maximum of 141 cases (Fig. 3). Observers were notified of the model’s performance on the validation dataset (not on the test dataset), including top-1 and top-2 accuracy and the confusion matrix.

**Fig. 4:**
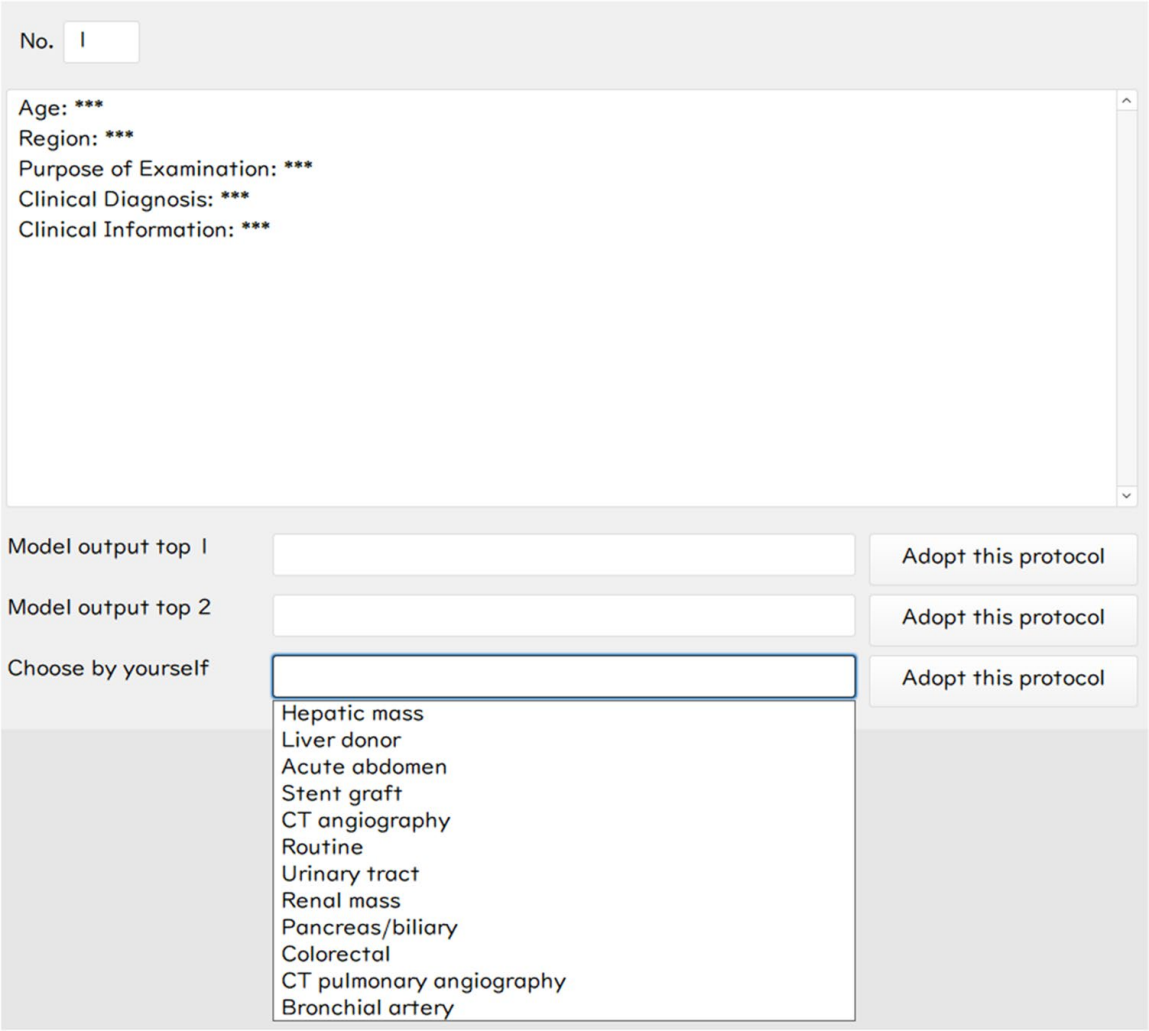
The user interface is used for observer performance tests. The figure shows the scenario with the assistance of a large language model. The top-1 and top-2 labels from the fine-tuned LLM’s output are provided above the dropdown list of all 12 protocols. The reader accepts a protocol by pressing the “Adopt this protocol” button

FileMaker Pro 18 Advanced (Claris International, Inc.) was used for the user interface to simulate a real-world input interface (Fig. 4). Only the clinical indication, age, and anatomic coverage of the requests were viewed, with 12 protocol choices provided, in the scenario without assistance from the fine-tuned LLM. Additionally, decision support information, specifically the top-1 and top-2 labels (class with the highest and the second-highest logits of the model’s output) from the fine-tuned LLM’s output, was provided for reference in the scenario with assistance. The labels assigned by the readers and their review times for each case were documented.

Bad protocoling causes excessive or inadequate tests, resulting in unnecessary radiation exposure or failure to address the clinical question; thus, we evaluated the clinical effect of protocol errors. We reviewed each case and categorized them into the following groups: “optimal,” where the protocol agreed with the reference standard; “suboptimal,” where the protocol was not optimal but did not significantly affect clinical outcomes; and “incorrect,” where the protocol noticeably compromised the examination quality.

### Statistical Analyses

R version 4.3.2 (https://www.r-project.org/) was used for statistical analyses. Readers were sub-categorized into radiologists and radiology residents for analysis. The McNemar test was utilized to compare the accuracy between radiologists and radiology residents with vs. without LLM. The Wilcoxon signed-rank test compared the macro sensitivity and the reading time between radiologists with and without LLM and between radiology residents with and without LLM. A *p*-value of < 0.05 indicated a statistically significant difference.

## Result

### Effect of Training Epochs on Model Performance in the Validation Dataset

**Fig. 5:**
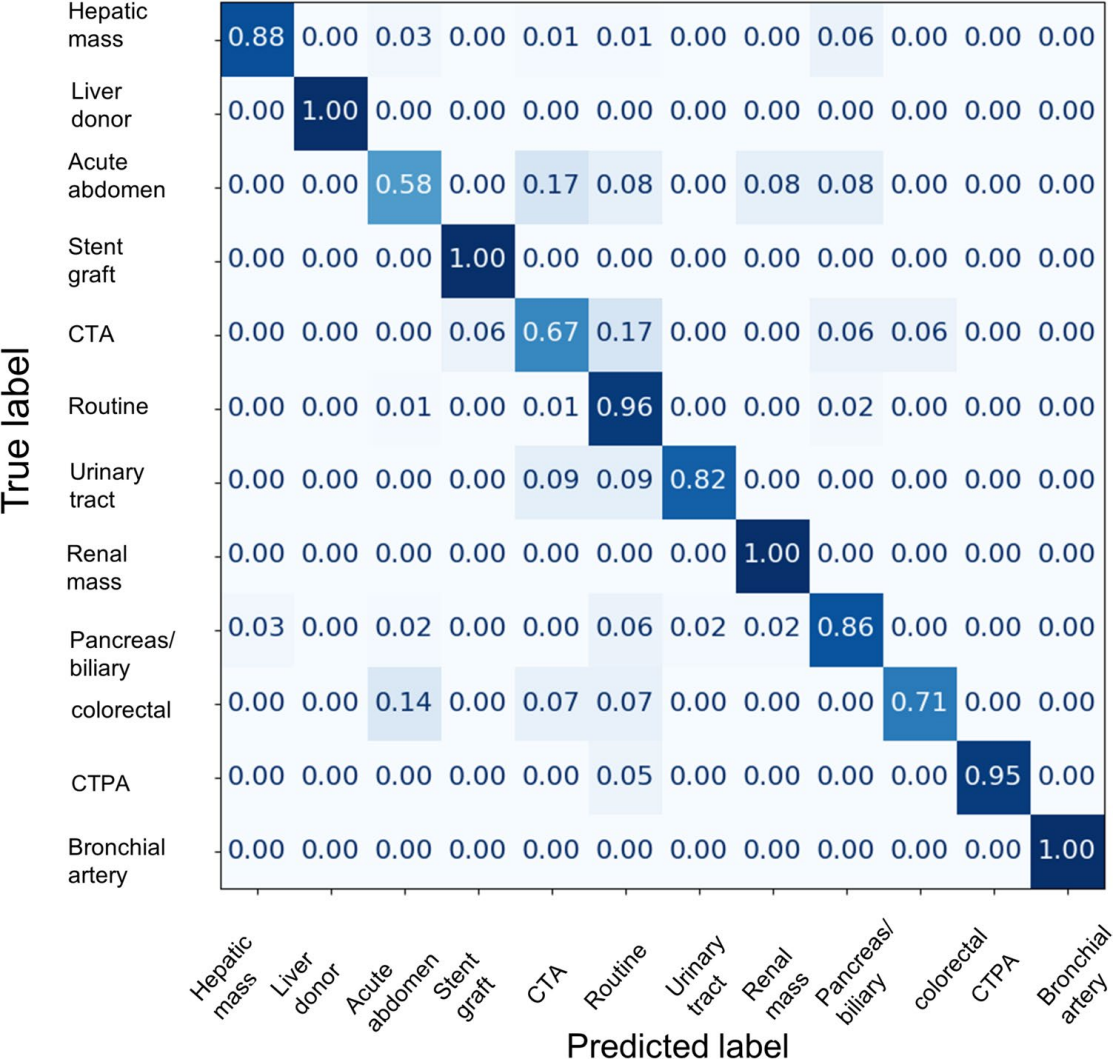
The image is a confusion matrix exhibiting the performance of a fine-tuned large language model in the validation dataset. The y-axis lists the true labels, whereas the x-axis demonstrates the labels predicted by the model. Each cell in the matrix contains a number that represents the proportion of instances where the predicted label corresponds to the true label. The diagonal cells, where the predicted labels match the true labels, indicate the class sensitivity. CTA, CT angiography; CTPA, CT pulmonary angiography

The macro sensitivity of the model revealed an increasing trend up to 6 epochs and reached an almost plateau at approximately 15 epochs, whereas weighted accuracy saturated at 3 epochs. The 95% confidence intervals for macro sensitivity and weighted accuracy were 0.908–0.915 and 0.769–0.804, respectively, with standard deviations of 0.0149 and 0.0774. The one trained for 6 epochs was the model with the best macro sensitivity, achieving top-1 and top-2 accuracies of 0.906 and 0.962, respectively, and the macro sensitivity of 0.869. Figure 5 illustrates the confusion matrix of the best model on the validation dataset.

### Standalone Performance of the Fine-Tuned Model in the Selected Test Dataset

**Fig. 6:**
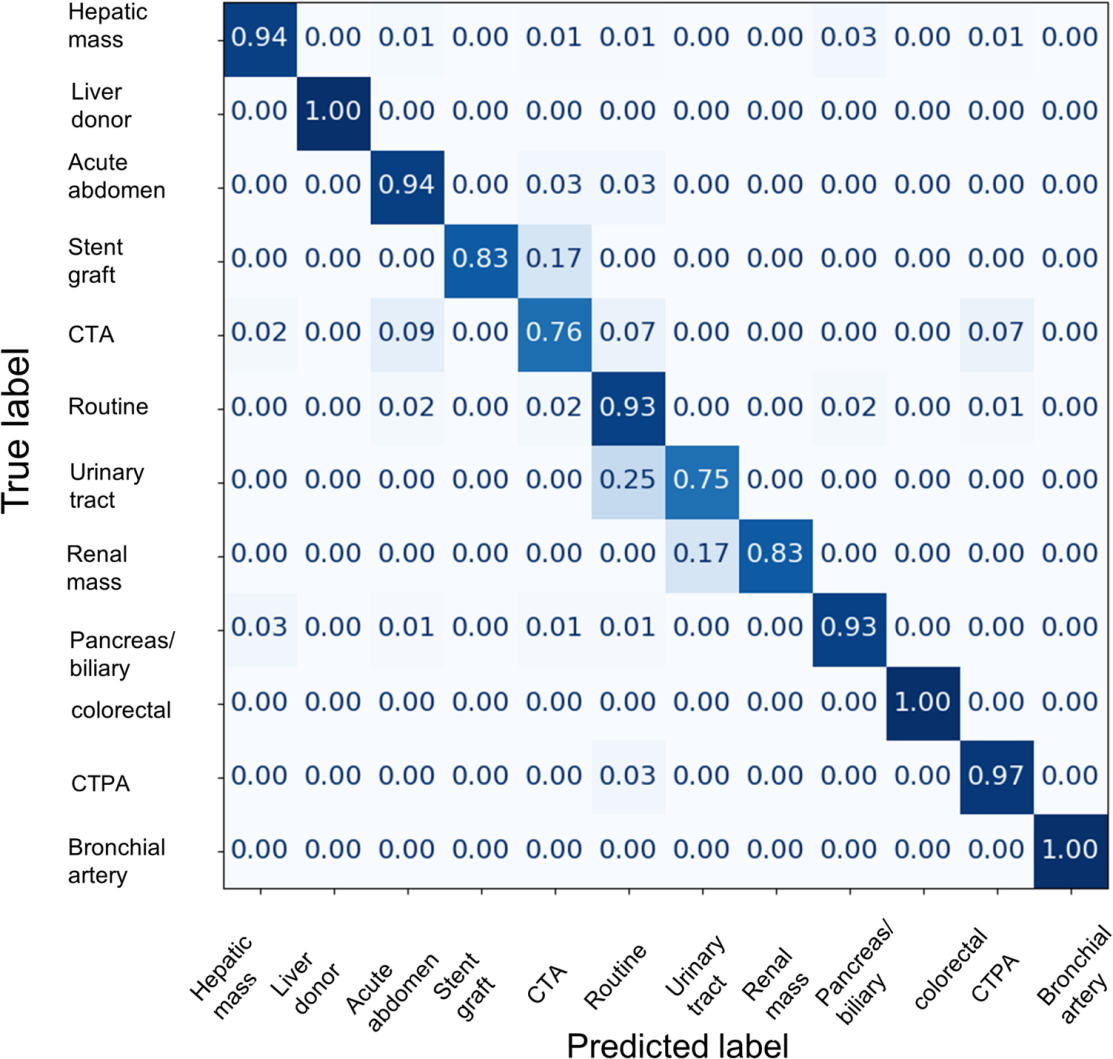
The image is a confusion matrix demonstrating the performance of a fine-tuned large language model in the test dataset. The y-axis lists the true labels, whereas the x-axis demonstrates the labels predicted by the model. Each cell in the matrix contains a number that represents the proportion of instances where the predicted label corresponds to the true label. The diagonal cells, where the predicted labels match the true labels, denote the class sensitivity. CTA, CT angiography; CTPA, CT pulmonary angiography

Top-1 and top-2 accuracy and macro sensitivity of the fine-tuned model in the selected test dataset (blocks A–H, 800 cases) were 0.923, 0.963, and 0.907, respectively. Figure 6 shows the confusion matrix of the best model on the selected test dataset. The reading time of the fine-tuned LLM was 314 s (0.35 s/case).

### Evaluation of the Fine-Tuned Model as a Clinical Decision Support System

#### Efficiency

**Fig. 7:**
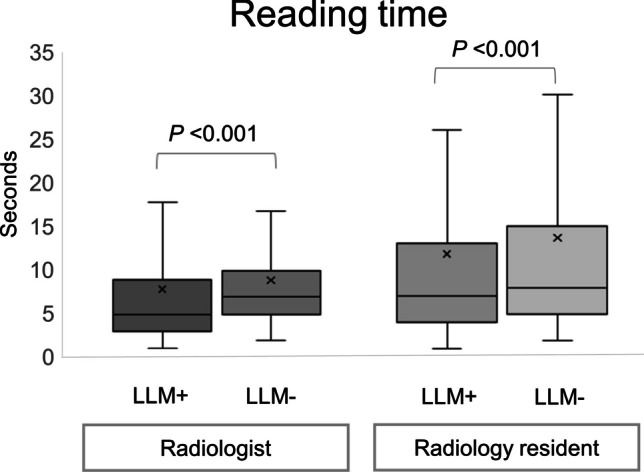
Effect of large language model (LLM) on reading time of CT protocoling for radiologists and radiology residents. The central line represents the median reading time for each group. The upper and lower edges of the box represent the 25th and 75th percentiles. The whiskers extend from the box to the smallest and largest values within 1.5 times the interquartile range from the 25th and 75th percentile, respectively. The “X” inside each box represents the mean reading time. The Wilcoxon signed-rank test was used for comparisons between radiologists with or without fine-tuned LLM. The *P-*value indicates the statistical significance of the difference in reading times between with LLM and without LLM. LLM + , with a large language model. LLM − , without a large language model

The combined reading time (blocks A–H) was 6314 and 7155 s for the radiologist and 9446 and 10,926 s for the radiology resident, with and without the fine-tuned LLM, respectively. This indicates a time reduction due to the fine-tuned LLM of 12% and 14% for radiologists and radiology residents, respectively. Figure 7 illustrates box plots of the reading time for each case with and without the LLM. The time reduction was statistically significant (*p* < 0.001) for both the radiologist and the radiology resident.

#### Effectiveness

The combined accuracy (blocks A–H) was 0.926 and 0.920 for the radiologist and 0.935 and 0.911 for the radiology resident, with/without the fine-tuned LLM, respectively (Table 3). These accuracies were comparable to those of the fine-tuned LLM alone (0.923). The accuracy for the radiology resident statistically significantly improved with the fine-tuned LLM (*p* = 0.02), whereas the change in the accuracy for the radiologist demonstrated no significant difference (*p* = 0.53).

**Table 3:** Weighted accuracy and macro sensitivity in the selected test dataset

	Radiologist			Radiology resident			LLM standalone
	With LLM	Without LLM	*p*-value	With LLM	Without LLM	*p*-value	
Weighted accuracy	0.926	0.920	0.53	0.936	0.913	0.02*	0.923
Macro sensitivity	0.935	0.888	0.03*	0.917	0.898	0.07	0.907

Comparisons of weighted accuracy between radiologists with or without fine-tuned LLM were performed using the McNemar test. Comparisons of macro sensitivity between radiologists with and without the fine-tuned LLM were performed using Wilcoxon’s signed-rank test, *LLM* large language model

^*^Statistically significant difference (*p* < 0.05)

### Evaluation of the Error Cases on Clinical Impact

The counts of optimal, suboptimal, and incorrect cases were 738, 22, and 40 for standalone LLM; 736, 28, and 36 for radiologists without LLM; 741, 23, and 36 for radiologists with LLM; 729, 16, and 55 for radiology residents without LLM; and 750, 19, and 31 for radiology residents with LLM, respectively (Table 4). The cases where the LLM was correct in its recommendation while radiologists or radiology residents incorrectly chose a different protocol were 18 and 16, respectively. Conversely, the cases where the LLM was incorrect in its recommendation and radiologists or radiology residents correctly overruled it were 36 and 39, respectively.

**Table 4:** Distribution of cases categorized as optimal (same as reference standard), suboptimal (different from reference standard but with less clinical impact), and incorrect (different from reference standard with certain clinical impact)

	Optimal	Suboptimal	Incorrect
Radiologist			
Without LLM	736	28	36
With LLM	741	23	36
Radiology resident			
Without LLM	729	16	55
With LLM	750	19	31
LLM (top-1)	738	22	40

*LLM* large language model

**Fig. 8:**
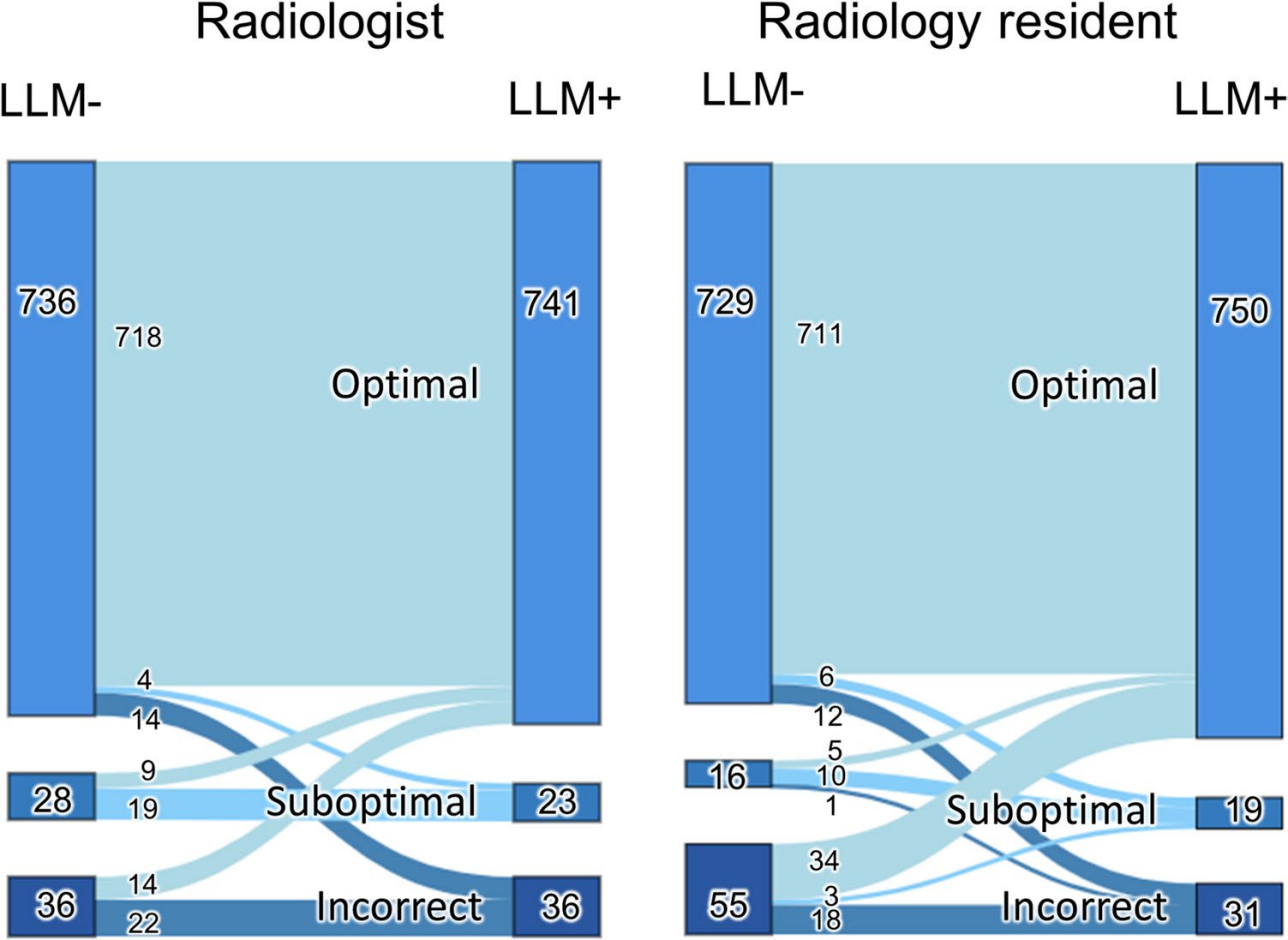
A diagram illustrating transitions across categories with and without a large language model (LLM). The numbers in the diagram represent the distribution and transitions of cases between optimal, suboptimal, and incorrect categories for radiologists and radiology residents with and without LLM. LLM + , with a large language model. LLM − , without a large language model

Figure 8 illustrates the distribution shift across categories (optimal, suboptimal, and incorrect) with and without the use of LLM among radiologists and radiology residents. The number of cases with incorrect protocols without LLM but optimal protocols with LLM for radiology residents was larger than that of optimal protocols without LLM but incorrect protocols with LLM (34 and 12 cases, respectively). Conversely, the number of cases with suboptimal protocols without LLM but optimal protocols with LLM for radiologists was slightly larger than that of optimal protocols without LLM but suboptimal protocols with LLM (9 and 4 cases, respectively).

The LLM struggled with prioritizing multiple clinical questions or multiple organs of interest and sometimes failed to capture the clinical picture because of uncommon medical terms or complex clinical histories regarding incorrect protocols in individual cases. A similar trend was observed in radiology residents. Specifically, among the cases where the LLM’s predictions were incorrect (47 in the validation set and 62 in the test set), there were 3 and 4 instances, respectively, where specific medical terms were not present in the training set. Additionally, some apparent careless mistakes were found in both radiology residents and radiologists, both with and without the LLM. Table 5 details incorrect cases in specific examples (#1 the LLM could mislead, #2 improved protocoling with the LLM, and #3 only the LLM made wrong labeling).

**Table 5:** Selected examples of the dataset

Type	Indication	Reader	Label
LLM could mislead protocoling	Age: 46; region: chest to pelvis; purpose of examination: inflammation; clinical diagnosis: suspected reactive arthritis; clinical information: the patient has experienced vomiting since January, diarrhea since February, and arthritis and fever since March, prompting further investigation for reactive arthritis. A fever of 38°, a C reactive protein level of 7, and right knee joint pain persist while there has been some improvement in symptoms with steroid treatment. The patient remains bedridden, and few results indicate deep vein thrombosis although the d-dimer level is elevated at 12. Please assess for the presence of thrombosis and investigate other sources of inflammation	Reference standard	Routine
		LLM	CT pulmonary angiography
		Radiologist, LLM − /LLM +	Routine/routine
		Radiology resident, LLM − /LLM +	Routine/CT pulmonary angiography
Improved protocoling with LLM	Age: 48; region: neck to pelvis; purpose of examination: tumor; clinical diagnosis: suspected chronic inflammatory demyelinating polyneuropathy; clinical information: progressive polyneuropathy over six months. Paraneoplastic syndrome is also a differential diagnosis. The investigation is intended to search for neoplastic lesions	Reference standard	Routine
		LLM	Routine
		Radiologist, LLM − /LLM	Routine/routine
		Radiology resident, LLM − /LLM	CT angiography/Routine
Only LLM made the wrong protocol	Age: 54; region: chest to the pelvis; purpose of examination: bleeding; clinical diagnosis: clinical information: the patient is attending our cardiology outpatient clinic for chronic thromboembolic pulmonary hypertension and visited the emergency department today at approximately 11:00 a.m. with hematemesis. Suspected alveolar hemorrhage, gastrointestinal bleeding, and superior mesenteric artery dissection have been noted. Contrast-enhanced CT aims to assess these conditions	Reference standard	CT angiography
		LLM	CT pulmonary angiography
		Radiologist, LLM − /LLM	CT angiography/CT angiography
		Radiology resident, LLM − /LLM	CT angiography/CT angiography

Actual input was in Japanese, while the translated examples provided are in English for easier overview, *LLM* large language model, *LLM* + with large language model, *LLM − *without large language model

## Discussion

The assignment of CT protocols is crucial to ensuring that exams are appropriately conducted. However, this process can be error-prone and time-consuming [8.9]. While today’s large language models hold promise for improving this, their impact on CT protocoling workflows remains unknown. This study developed a fine-tuned large language model for CT protocoling and assessed its efficacy within the simulated radiological workflow. The top-1 and top-2 accuracy and macro sensitivity of the fine-tuned large language model were 0.923, 0.963, and 0.907, respectively, with a reading time of 0.39 s/case. Both radiology residents and radiologists demonstrated improved accuracy and macro sensitivity when using the clinical decision support system. A statistically significant improvement was observed in the accuracy of radiology residents (*p* = 0.02) and in the macro sensitivity of radiologists (*p* = 0.03). The reading time significantly declined by 14% and 12% for radiology residents and radiologists, respectively.

Previous studies have investigated various algorithmic approaches for CT or MRI imaging protocol assignment [5–7, 10–17]. This study used the BERT model for classification tasks for several reasons. First, BERT is popular for its deep contextual word embeddings, which are essential for processing complex indications, among several language models. Indeed, three studies reported on its use for CT protocoling [5–7], and three studies reported for MRI protocoling [5, 6, 14], all of which demonstrated promising results. Specifically, Talebi et al. [14] achieved high performance with the BERT-based model (accuracy: 0.93), which was comparable or slightly inferior to humans (accuracy 0.97) and superior to k-nearest neighbors, random forest, gradient booted machines, and deep neural networks (F1 score 0.70, 0.77, 0.84, 0.85, respectively). Second, fine-tuned BERT models can be developed with relatively small datasets and simple programming, improving their customizability and flexibility. Our study revealed high accuracy in the fine-tuned LLM. This performance was comparable to previously reported models [5, 14], although the direct comparison was challenging due to differences in modalities, input exam information (sex, age, and anatomic coverage), language, and the number of classification classes. The fine-tuned LLM struggled with prioritizing various clinical queries or organs of interest and sometimes failed to capture the clinical context due to uncommon medical terms or complex clinical histories. This trend is congruent with results from previous studies on the use of BERT models for MRI protocoling [14].

Models frequently become biased toward the majority class, causing suboptimal classification and frequent misclassification of minority class instances, due to data imbalance. We employed cost-sensitive learning methods to address this issue [21, 22]. Weighted accuracy may fail to adequately evaluate less frequent categories in evaluating the performance of models trained on imbalanced datasets. Therefore, we selected macro averaged sensitivity as our metric for selecting the best model [21], as it provides equal weighting to the performance of each class. The fine-tuned LLM exhibited high accuracy and satisfactory macro sensitivity, achieving a minimum sensitivity of 0.75.

Our results revealed that, despite considerations inherent in clinical decision support system, our model improved the radiological workflow in both efficiency and effectiveness. For efficiency, the reading time decreased by 14% for radiology residents and 12% for radiologists. This reduction may partly be associated with the LLM’s suggestions of providing additional materials for judgment, thereby reducing the time spent deliberating between multiple protocols. Additionally, selecting from long lists of protocols on the interface is time-consuming. With the LLM, this burden was alleviated because the top two protocols were presented in a specific corner of the interface. This factor exhibited a larger effect when protocol lists were longer. Regarding effectiveness, macro sensitivity was 0.935 and 0.888 for the radiologist and 0.917 and 0.898 for the radiology resident, demonstrating a statistically significant improvement with the fine-tuned LLM for the radiologist (*p* = 0.03) and a statistically insignificant change for the radiology resident (*p* = 0.07). The improvement in macro sensitivity for radiologists using the LLM was statistically significant, while the improvement in accuracy was not. This discrepancy was because the accuracy for the less frequent category improved, while that of the frequent category did not, suggesting the potential of the LLM to help assign less frequent protocols correctly. This effect would be more advantageous when there are more rare specialized protocols.

Regarding error cases, the use of the LLM decreased incorrect assignments by radiology residents, whereas this trend for radiologists was not apparent. This indicates that a fine-tuned LLM could be particularly effective in preventing incorrect protocols among those with less experience. Conversely, suboptimal cases assigned by radiologists were more appropriate with the LLM. This improvement indicates that the LLM helps align protocols more closely with institutional standards because the distinction between suboptimal and optimal protocols frequently depends on institutional rules rather than medical knowledge in this study. Utilizing the LLM could lead to more standardized and consistent protocol assignments, which is important for ensuring the quality of imaging examinations [8]. Although the proportion of improvement was not substantial, the impact is not negligible considering the vast number of contrast-enhanced CT exams performed daily, all of which require protocol assignments.

This study has some limitations. First, our training datasets were relatively small. However, the performance was comparable to previous reports, which was satisfactory to assess the effect of the clinical decision support system on radiology workflow. Additionally, our dataset was from a single institution with institution-specific protocol lists, thereby limiting generalizability. However, our study focused on investigating LLMs in real-world practice, establishing tailor-made LLMs suitable as they matched our workflow. Second, only contrast-enhanced CT exam indications were included, and unenhanced CT indications were excluded because our institution’s practice leaves the decision to use contrast up to the ordering physician. We hypothesized that protocoling for contrast-enhanced CTs warrants more specialized medical and radiological knowledge compared to unenhanced CTs, thereby proposing more difficult and complex tasks to LLM. Third, rare protocols were excluded due to insufficient training data, thereby potentially limiting the breadth of protocols represented in clinical practice. Fourthly, our protocoling design included limited information (age, anatomic coverage, and exam indication) while omitting potentially relevant data, including laboratory results, prior imaging results, and previous imaging exams. This could partially explain why protocoling times per case were shorter than expected. Fifth, the BERT model used in this study was not specialized for the biomedical domain, which may pose challenges in understanding domain-specific medical terms. To address this, we ensured that the fine-tuning dataset included diverse clinical indications and relevant medical terminology. While there were a few incorrect predictions where specific medical terms were absent from the training set, these instances were rare and had minimal impact on the overall performance. This suggests that the limitations of a general-domain BERT model were largely mitigated by the diversity of the training dataset. Nonetheless, unfamiliar medical terms in the validation and test sets could still affect the model’s performance. Future research could benefit from pre-training on a specialized medical corpus [23]. Lastly, we aimed to limit protocoling tasks to 400 cases per session because the reader’s fatigue deteriorates data quality [24], but this workload was more burdensome compared to their usual workflow (100 cases per day for contrast-enhanced CT exams), potentially causing careless mistakes.

In conclusion, the standalone fine-tuned LLM exhibited high performance with an accuracy of 0.923, comparable to that of both radiologists and superior to radiology residents. It improved accuracy among radiology residents and macro sensitivity among radiologists when used as a clinical decision support system. Moreover, it reduced the reading time by 14% and 12% for radiology residents and radiologists, respectively, emphasizing the practical benefit of using the fine-tuned LLM as a clinical decision support tool.

## Supplementary Information

Below is the link to the electronic supplementary material.

Supplementary file1 (PY 5 KB)

## Data Availability

The code used for fine-tuning is provided in the supplemental material. The datasets generated and analyzed during the current study are available from the corresponding author upon reasonable request.
